# Hyaluronan-carnosine conjugates inhibit Aβ aggregation and toxicity

**DOI:** 10.1038/s41598-020-72989-2

**Published:** 2020-09-29

**Authors:** Valentina Greco, Irina Naletova, Ikhlas M. M. Ahmed, Susanna Vaccaro, Luciano Messina, Diego La Mendola, Francesco Bellia, Sebastiano Sciuto, Cristina Satriano, Enrico Rizzarelli

**Affiliations:** 1 Department of Chemical Sciences, University of Catania, A. Doria 6, 95125 Catania, Italy; 2 Inter-University Consortium for Research on the Chemistry of Metal Ions in Biological Systems, C. Ulpiani, 27, 70126 Bari, Italy; 3 Institute of Crystallography, CNR, P. Gaifami 18, 95126 Catania, Italy; 4 Fidia Farmaceutici, 96017 Noto, SR Italy; 5 Department of Pharmaceutical Sciences, University of Pisa, Bonanno Pisano 12, 56126 Pisa, Italy

**Keywords:** Chemical modification, Peptides

## Abstract

Alzheimer’s disease is the most common neurodegenerative disorder. Finding a pharmacological approach that cures and/or prevents the onset of this devastating disease represents an important challenge for researchers. According to the amyloid cascade hypothesis, increases in extracellular amyloid-β (Aβ) levels give rise to different aggregated species, such as protofibrils, fibrils and oligomers, with oligomers being the more toxic species for cells. Many efforts have recently been focused on multi-target ligands to address the multiple events that occur concurrently with toxic aggregation at the onset of the disease. Moreover, investigating the effect of endogenous compounds or a combination thereof is a promising approach to prevent the side effects of entirely synthetic drugs. In this work, we report the synthesis, structural characterization and Aβ antiaggregant ability of new derivatives of hyaluronic acid (Hy, 200 and 700 kDa) functionalized with carnosine (Car), a multi-functional natural dipeptide. The bioactive substances (HyCar) inhibit the formation of amyloid-type aggregates of Aβ_42_ more than the parent compounds; this effect is proportional to Car loading. Furthermore, the HyCar derivatives are able to dissolve the amyloid fibrils and to reduce Aβ-induced toxicity in vitro. The enzymatic degradation of Aβ is also affected by the interaction with HyCar.

## Introduction

Senile plaques and neurofibrillary tangles (NFTs) characterize the brains of Alzheimer’s disease (AD)-affected patients, and the brains of these patients also show loss of synapses and neurons. The major components of plaques are amyloid peptides (Aβs), while NFTs contain phosphorylated tau proteins^1^.

According to the amyloid cascade hypothesis^2^, increases in extracellular Aβ levels give rise to different amyloid species, namely, protofibrils, fibrils and oligomers^3,4^, with oligomers being more toxic than the other species for cells^5^.

Aβ peptides are generated from the amyloid precursor protein through the cleavage activity of some membrane-bound proteases^6^. Between the main isoforms (Aβ_40_ and Aβ_42_), Aβ_42_ is known to be more toxic and represents the major component of Aβ plaques^7^.

**Figure 1:**
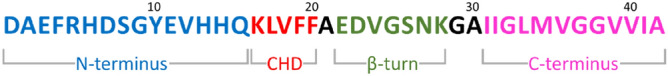
Aβ_42_ peptide regions. The N-terminal region (segment 1–15), central hydrophobic domain (CHD) (segment 16–20), β-turn region (segment 22–27), and C-terminal region (segment 31–40/42) are outlined.

The intrinsically disordered nature of Aβ peptides and their high propensity to aggregate make it difficult to achieve structural characterization of these peptides^8^; circular dichroism spectra show that these peptides adopt a mixture of random coil and α-helical structures in aqueous solution and an increased β-sheet population in oligomeric species and fibrils^9^. The formation of oligomeric species and fibrils is related to the specific Aβ amino acid sequence. The Aβ peptide is an amphipathic polypeptide with several different regions, including a hydrophilic N-terminus^10–13^, central hydrophobic domain (CHD)^14,15^, turn regions^16,17^, and hydrophobic C-terminus^18^ (Fig. 1). These different regions influence Aβ aggregation in different ways^16^.

Dimer formation due to the interactions spanning residues Lys16-Phe20 (Fig. 1), i.e., the CHD domain, is the proposed first step of oligomerization with Aβ self-recognition that drives the monomer hairpins in an antiparallel β-sheet conformation. Furthermore, the His13-Lys16 (HHQK) region of Aβ_42_ at the N-terminus is very important in oligomerization and fibril formation and accounts for the neurotoxicity of the Aβ peptide^19–21^.

Therefore, approaches that inhibit toxic Aβ species formation have been adopted, and the identification of different antagonists of Aβ self-assembly has been reported, including of small molecules^22–24^, antibodies^25^, molecular chaperones^26^, and linear and cyclic peptides^27–29^.

However, most of the efforts have recently been focused on multi-target ligands^30^ to address the numerous events that occur concurrently with the onset of the disease, such as oxidative stress and metal dyshomeostasis^31,32^.

Recent findings indicate that a natural water-soluble dipeptide, β-alanyl-L-histidine (carnosine, Car), and some homologues, such as homocarnosine and anserine, possess protective properties against AD^33–35^. Car shows inhibitory activity against the toxicity and aggregation of amyloidogenic species^36–38^. This dipeptide protects rat brain cells from Aβ toxicity^39,40^ and reduces Aβ aggregation in vivo, improves AD-related mitochondrial dysfunction and restores hippocampal and cerebral complexes in AD-transgenic mice^33^. Furthermore, this highly soluble dipeptide does not form fibril-containing species; on the contrary, it attenuates the in vitro fibrillogenesis of Aβ_42_^37,41^, (i) resulting in dose-dependent arrest of Aβ_42_ polymerization, (ii) promoting the production of shorter fibrils and globular aggregates, (iii) reducing the mean fibril length and (iv) halting the growth of pre-existing fibrils^41^.

Car shows different advantageous functions, such as anti-glycation and antioxidant functions, maintenance of pH balance, and chelation of metals, including Zn^2+^ and Cu^2+^ ^42,43^. Furthermore, supplementation therapy with Car and anserine is reported to be effective at improving cognitive impairment in the elderly^44^.

The action serum- and tissue-distributed Carnosinases^45^ severely limits the potential pharmacological use of Car. For this reason, the dipeptide has been chemically conjugated with other endogenous compounds in order to reduce the peptide hydrolysis and improve its multifunctional properties^46^. Novel bifunctional Car derivatives have been proposed as inhibitors of Aβ aggregation, copper chelation and antioxidant molecular entities^47–49^.

Hyaluronic acid^50^ (Hyaluronan Hy) is a nonsulphated glycosaminoglycan (GAG) that contains a disaccharide repeat structure [-4-d-glucuronic acid-β1-3-*N*-acetylglucosamine-β1-]_n_. This polyanionic linear compound is widely distributed in mammalians. The biomechanical and biochemical properties of Hy make this polymer able to perform several physiological functions, such as the hydration and turgidity maintenance of tissue, the preservation of the extracellular matrix structure, the regulation of innate immunity, the protection and lubrication of joints^50,51^. Due to this versatility, Hy represents a promising bio-indicator of pathophysiology and inflammation. Therefore it has consequently been used for disease-specific diagnostics^52,53^. The wild-type Hy has a molecular weight (MW)^54^ in the range of 10^5^–10^7^ Da, and the size is a critical characteristic of the molecule’s function in vivo. High-MW Hy (> 1000 kDa) is extremely viscous and exhibits apparent anti-inflammatory and immunosuppressive properties^55^, whereas low-MW Hy (smaller than 500 kDa) promotes the cytokines release from macrophages^56^.

**Figure 2:**
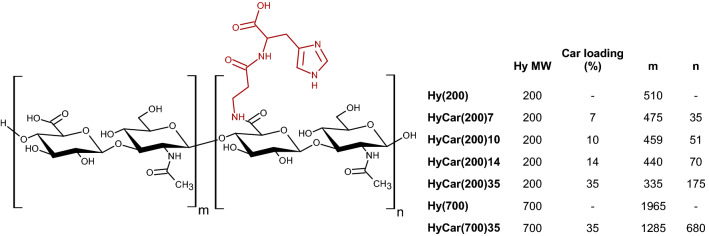
Schematic structure and list of the HyCar derivatives and their parent polymeric compounds. The average number of repeat units conjugated or not to Car (n and m, respectively) was calculated on the basis of the structural characterization (see “Experimental” section). The Car moieties are randomly distributed throughout the hyaluronan sequence.

Herein, we describe the design and synthesis of a new Car Aβ aggregation inhibitor, HyCar, through combination of a natural dipeptide (Car) and hyaluronic acid (Hy) of two different molecular weights (200 and 700 kDa) and different loading amounts of Car (Fig. 2). In addition, we report the ability of Hy to protect Car from the proteolytic action of carnosinase^60,73^. The capacity of the new HyCar derivatives to inhibit the formation of amyloid-type aggregates of Aβ was tested by using fluorescence and atomic force microscopy (AFM) measurements. Finally, the effects of the HyCar-Aβ interaction on (i) the enzymatic hydrolysis of Aβ and (ii) the cellular cytotoxicity due to Aβ oligomers were also assayed.

## Results

### Synthesis of Hy conjugates with Car

Several methods of conjugating bioactive substances to hyaluronic acid have been reported in the literature^58^. We did not use any spacer linker to increase the resistance of the conjugate to carnosinase activity^60^; therefore, we chose to link hyaluronic acid to Car through an amide bond between the carboxylic group of the d-glucuronic unit of the polysaccharide and the N-terminal amino group of Car. Accordingly, hyaluronic acid-Car conjugates were prepared following a synthetic route that involves the activation of the Hy-linked carboxylate groups and their subsequent conversion into amide bridges by reaction with the amino group of carboxyl-protected Car. The final products differ in terms of MW of the starting Hy (200 or 700 kDa) and Car loading (7, 10, 14, 35%), i.e., the percentage of D-glucuronic units drafted by the Car residues.

Subsequently, NMR analysis of the HyCar conjugates was performed to ascertain the chemical conjugation and to calculate the Car loading percentage of each new HyCar derivative. Finally, the medium molecular weight (MMW) and the intrinsic viscosity (I.V.) (Supplementary Table S1) of each conjugate were determined, and the results were in accordance with those obtained from the NMR analysis^59^. The greater the loading percentage of Car was, the higher the MMW, as expected. Moreover, Car conjugation clearly affects the final viscosity: all the HyCar derivatives showed an I.V. value lower than that of the parent polymer compounds. This difference clearly increased as the MMW increased after Car derivatization.

The structural characterization also confirmed that no fragmentation of the polysaccharide skeleton occurred as a consequence of the synthesis process.

### Carnosinase resistance

**Figure 3:**
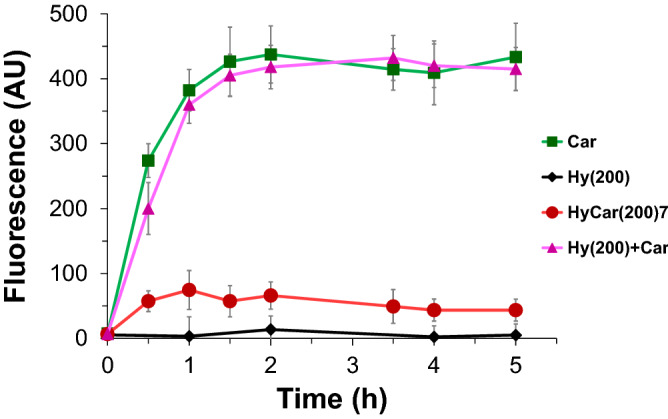
Enzymatic activity towards HyCar(200)7 and its parent compounds. The fluorescence intensity (proportional to the histidine content) reported over time due to the action of carnosinase on HyCar(200)7, carnosine (Car), hyaluronic acid (Hy) and the mixture of the latter two.

Carnosinase (CN1) is a dipeptidase that is present in blood serum and degrades histidine-containing dipeptides such as Car^60^. Therefore, to assess the stability of the HyCar conjugates towards the enzyme carnosinase, the following experiments were carried out by using HyCar(200)7 as a model substrate. The time-dependent stability of this conjugate towards CN1, compared with that of Car and a mixture of Car plus Hy, was determined by incubating each of these substances (or mixtures) with the enzyme. The histidine released during each incubation period was determined by a fluorometric assay^60^. The stability of HyCar towards the action of carnosinase was approximately nine times greater than that of Car used either by itself or in a mixture with hyaluronic acid. The results of this test are shown in Fig. 3.

HyCar(200)7 is the smaller HyCar derivative tested in this work. To verify whether the MW of the hyaluronan moiety of the HyCar conjugates (or their Car loading) could have any effect on carnosinase resistance, we also tested the CN1-mediated degradation of the larger HyCar derivative (HyCar(700)35). The results (Figure S1) clearly show that HyCar(700)35 is also resistant to enzymatic hydrolysis of the Car moiety.

### Antiaggregation activity

The amyloid-type aggregation of Aβ starts with the formation of soluble and toxic oligomers; their dimensions increase during the process, and fibrillary and insoluble structures are formed at the end of this pathological pathway. Reducing or preventing the aggregation process of Aβ is one of the main challenges associated with attenuating or preventing the onset of AD. Therefore, all HyCar derivatives were tested for the in vitro self-induced aggregation of Aβ_42_ by using thioflavin T (ThT), a fibril-sensitive dye.

**Figure 4:**
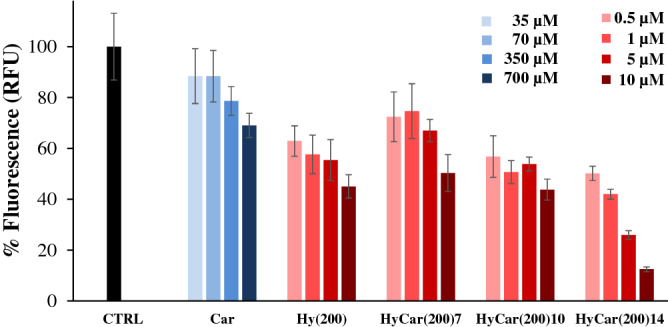
Final antiaggregant activity of HyCar derivatives towards the amyloid-type aggregation of Aβ. Relative fluorescence values of the samples containing Aβ_42_ (15 µM) alone (CTRL), in the presence of the Hy(200) derivatives of Car (HyCar(200)7, HyCar(200)10, HyCar(200)14) or their parent compounds (Hy(200) or Car), incubated at 37 °C for 40 h. The concentration of Hy(200) and that of its Car conjugates (HyCar(200)7, HyCar(200)10, HyCar(200)14) varied from 0.5 to 10 µM, whereas the concentration range of Car (35–700 µM) is equivalent to the amount of Car in the samples containing HyCar(200)14.

As a first step, the extent of dose-dependent aggregation of the HyCar(200) derivatives and their parent compounds was monitored at the end of the incubation period (40 h). As reported in Fig. 4, the maximum ThT fluorescence variation due to amyloid aggregation was significantly reduced in the presence of Hy(200). In contrast, Car alone did not show any significant effect on the formation of ThT-sensitive species at the lower concentrations used (0.5 and 1 µM). However, both the underivatized Hy(200) polymer and the dipeptide (Car) were clearly able to partially inhibit the total amyloid species formation in a dose-dependent manner. The concentrations of Car were equivalent to the level of Car units in the samples containing HyCar(200)14.

HyCar(200) derivatives inhibit the formation of amyloid species, and the decrease in fluorescence with respect to that of Aβ alone (CTRL sample) is directly proportional to their concentration. Interestingly, the higher the Car content covalently linked to the Hy(200) scaffold was, the better the inhibition activity. As the main result, HyCar(200)14 showed the best performance among the HyCar(200) derivatives in terms of reducing the amyloid-type aggregation of Aβ_42,_ and the higher concentrations of this compound yielded a final fluorescence increment as low as 10% compared to that shown by Aβ_42_ alone.

Then, kinetic measurements of amyloid aggregation were carried out in order further study the antiaggregant capabilities of the HyCar(200) derivatives. The maximum fluorescence gain (*F*_*max*_* – F*_*0*_) is proportional to the aggregation extent of Aβ, whereas the lag time (*t*_*lag*_) value represents the starting point of the amyloid-type aggregation of Aβ. As a consequence, the lower *F*_*max*_* – F*_*0*_ is and the higher *t*_*lag*_ is, the better the antiaggregant activity.

**Figure 5:**
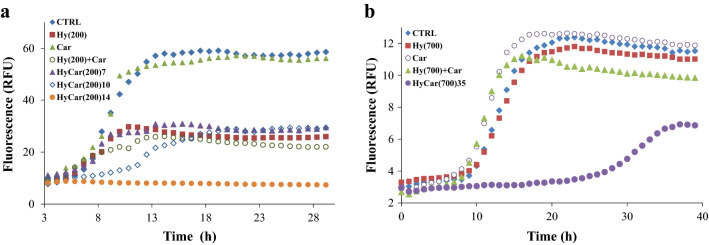
Kinetic trends of amyloid aggregation in the presence of HyCar derivatives. Kinetic profiles of Aβ_42_ aggregation alone (CTRL, 15 µM), incubated with the **(a)** Hy(200) derivatives of Car (HyCar(200)7, HyCar(200)10, HyCar(200)14) or their parent compounds (Hy(200), Car or a mixture) or **(b)** with the Hy(700) derivative of Car (HyCar(700)35), their parent compounds (Hy(700), Car or a mixture). The concentrations of the tested compounds were as follows: **(a)** 10 µM (Hy(200), HyCar(200)7, HyCar(200)10, HyCar(200)14), 700 µM (Car), **(b)** 0.4 µM (Hy(700), HyCar(700)35) or 270 µM (Car). The concentration values of Hy and Car in the Hy(200) + Car mixture are equivalent to the amount of Hy and Car units in the sample containing HyCar(200)14, whereas the concentration values of Hy and Car in the Hy(700) + Car mixture are equivalent to the amount of Hy and Car units in the sample containing HyCar(700)35.

When ThT is incubated with Aβ_42_, the amyloid-type aggregation mechanism results in a sigmoid-shaped fluorescence response (Fig. 5a). The lag phase lasts 5.9 h (Supplementary Table S2), and the maximum fluorescence gain (*F*_*max*_ − *F*_*0*_) reaches a value of 51.2.

For the treatment with Car, the kinetic parameters of the self-induced amyloid aggregation do not significantly change. When Hy(200) or a mixture of Hy(200) and Car was co-incubated with Aβ, the corresponding *F*_*max*_ − *F*_*0*_ values (17.4 and 16.4, respectively) were significantly lower than those fitted in the case of Aβ alone. Conversely, Hy(200) and Hy(200) + Car do not show any effect on the lag phase of the amyloid aggregation process. For HyCar(200)7, the fitted kinetic parameters do not significantly differ from those obtained in the case of the Hy(200) + Car mixture. HyCar(200)10 is able to delay amyloid aggregation with respect to HyCar(200)7. HyCar(200)14 greatly outperforms the antiaggregant activity exhibited by the Hy(200) derivatives with lower Car loading (HyCar(200)7 and HyCar(200)10). The ThT fluorescence signal over time becomes almost flat and thus cannot be properly fitted. The data reveal that the activity of HyCar(200)14 is not the sum of the antiaggregant activity of the parent compounds (Hy(200) and Car). The conjugation between the Hy(200) polymer and Car markedly improves the inhibitory effect of these systems with synergistic effects.

Car was conjugated to Hy(200) until 35% polymer loading was achieved. Both the kinetic profile of the antiaggregant activity and the dose-dependent effect of HyCar(200)35 do not differ from those of HyCar(200)14 (data not shown).

The amyloid aggregation assay was also used to test the Car derivative with Hy(700) (HyCar (700)35). The molar concentration range used for the Hy(700) derivatives (0.4 µM) was lower than that tested for activity evaluation of the Hy(200) derivatives (10 µM) because the former compounds are less soluble than the latter ones. Compared to the kinetic trend for amyloid aggregation shown by Aβ_42_ (Fig. 5b, CTRL sample), neither Hy(700) and Car (tested individually) nor the Hy(700) + Car mixture affected the lag phase of the process, although the presence of the latter slightly decreased the *F*_*max*_ − *F*_*0*_ value, while Hy(700) did not alter Aβ_42_ fibril formation (Supplementary Table S3).

On the other hand, the *F*_*max*_ − *F*_*0*_ value decreased to 4.4 and the lag phase was lengthened to 24.5 h when HyCar(700)35 was tested.

The excellent antiaggregation effect of HyCar(700)35 on amyloid formation was evaluated for dose dependence (Supplementary Figure S2, Table 1). Both the *F*_*max*_ − *F*_*0*_ and *t*_*lag*_ values progressively changed as a function of the HyCar(700) derivative amount, thus confirming the effect that Car derivatization of Hy(700) has on the antiaggregant activity.

**Table 1:** Kinetic parameters related to the aggregation of Aβ_42_ alone (CTRL) or incubated with HyCar(700)35 (20–400 nM).

	CTRL	HyCar(700)35 20 nM	HyCar(700)35 40 nM	HyCar(700)35 200 nM	HyCar(700)35 400 nM
F_max_^.^− F_0_	8.8 ± 0.5	6.8 ± 0.4	7.3 ± 0.2	4.8 ± 0.3	4.4 ± 0.2
*t*_*lag*_	9.8 ± 0.3	14.9 ± 0.4	19.1 ± 0.4	26.1 ± 0.5	24.5 ± 0.8

Overall, HyCar(200)14 and HyCar(700)35 show a prominent effect in delaying the formation and reducing the amount of amyloid-type aggregated forms of Aβ_42_.

We also evaluated the activity of Hy(200), Hy(700) and different Car conjugates on the dissolution of preformed Aβ_42_ fibrils (Supplementary Figure S3). After treatment with HyCar(200)14, ThT fluorescence was reduced by only 20%, whereas twice that value (40%) was observed when the amyloid fibrils were treated with HyCar(700)35.

The ability to dissolve the ThT-sensitive amyloid forms was also shown by Hy(700) and by a mixture of Hy(700) and Car, though the effect was significantly weaker than that exhibited by HyCar(700)35. This finding indicates that the covalent conjugation between Hy(700) and Car clearly results in a gain-of-function effect of both the Aβ antiaggregation and disaggregation activities.

### Enzymatic hydrolysis of Aβ

The non-covalent interactions between the HyCar derivatives and the amyloid peptide could also have an effect on the clearance of Aβ. Insulin-degrading enzyme (IDE)^61^ is a zinc metalloprotease that catalyses the hydrolysis of several polypeptides, such as insulin, IAPP, bradykinin, glucagon and Aβ.

For this reason, the IDE-mediated hydrolysis of Aβ_28_ was also assayed in the presence HyCar(700)35, the HyCar derivative with the highest antiaggregant activity among all the newly synthesized HyCar conjugates. Aβ_28_ was chosen as a model peptide for Aβ_42_ because it not only encompasses the N-terminus and CHD regions important for the oligomerization process but also shows an aggregation rate lower than that of Aβ_42_; these features make Aβ_28_ a suitable substrate for the time scale of the enzymatic assay.

The main purpose of this assay was to both further prove the HyCar-Aβ interaction and obtain indirect information about the portions of the Aβ sequence involved in the non-covalent interaction with the HyCar derivatives.

**Figure 6:**
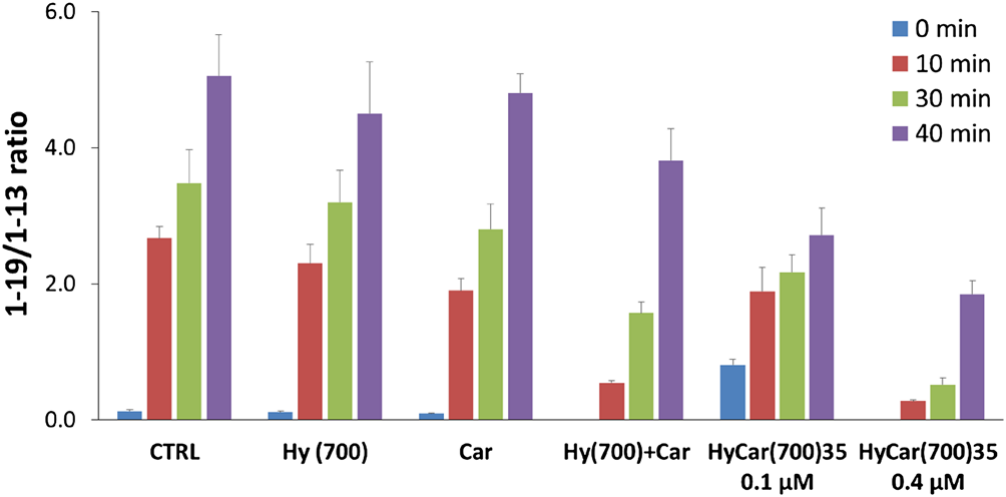
IDE-dependent hydrolysis of Aβ in the presence of HyCar(700)35 or its parent compounds. Time-dependent extent of IDE-mediated hydrolysis of Aβ_28_ alone (CTRL) or in the presence of HyCar(700)35 (0.1 and 0.4 µM), Hy(700) (0.4 µM), Car (270 µM) or a mixture of Hy and Car (Hy + Car, 0.4 and 270 µM, respectively). The concentration values of Hy and Car are equivalent to the amount of Hy and Car units in the sample containing 0.4 µM HyCar(700)35.

As reported in Supplementary Figure S4, Aβ alone (CTRL) was hydrolysed by IDE, mainly forming 1–13, 1–19, 1–15 and 5–17 hydrolytic peptides. Their normalized intensity changed during the reaction, and after 40 min, the hydrolytic pattern was very different from that detected a few min after the reaction began. In particular, 1–13 was the main fragment at the beginning of the reaction, whereas the intensity of 1–19 exceeded that of all the other hydrolytic peptides as early as 10 min after the reaction started. IDE-mediated digestion was also monitored in the presence of HyCar(700)35 (0.1 and 0.4 µM) or the parent compounds (Hy and Car), alone or in a mixture (Hy + Car). Hy and Car do not change the hydrolytic pattern of the amyloid substrate. Both the HyCar derivative and the mixture significantly affect the kinetic trend of the hydrolytic pattern. To better describe this effect, the ratio between the intensity of the main Aβ fragments (1–19 and 1–13) is reported in Fig. 6, showing the time-dependent effect of IDE-mediated hydrolysis on the different Aβ domains.

The 1–19/1–13 ratio rapidly increased when Aβ was incubated with IDE (CTRL). The same trend was observed when Hy or Car was co-incubated with the substrate. The presence of HyCar(700)35 at the lowest dose tested (0.1 µM) significantly slowed the formation and hydrolysis of 1–19 and 1–13, respectively, thus reducing the ratio 1–19/1–13. This effect was more prominent when the concentration of HyCar was higher (0.4 µM). This dose-dependent effect confirms once more the non-covalent interaction between HyCar(700)35 and the amyloid peptide. Aβ hydrolysis was also tested with a mixture of Hy and Car. Additionally, in this case, IDE-mediated hydrolysis was altered, but the effect was clearly milder than that of 0.4 µM HyCar(700)35.

### AFM measurements

To characterize the morphological features of the aggregated amyloid forms during Aβ aggregation as a function of the Hy derivatives, samples from the aggregation reactions (Supplementary Figure S5) were analysed by using Atomic Force Microscopy (AFM).

**Figure 7:**
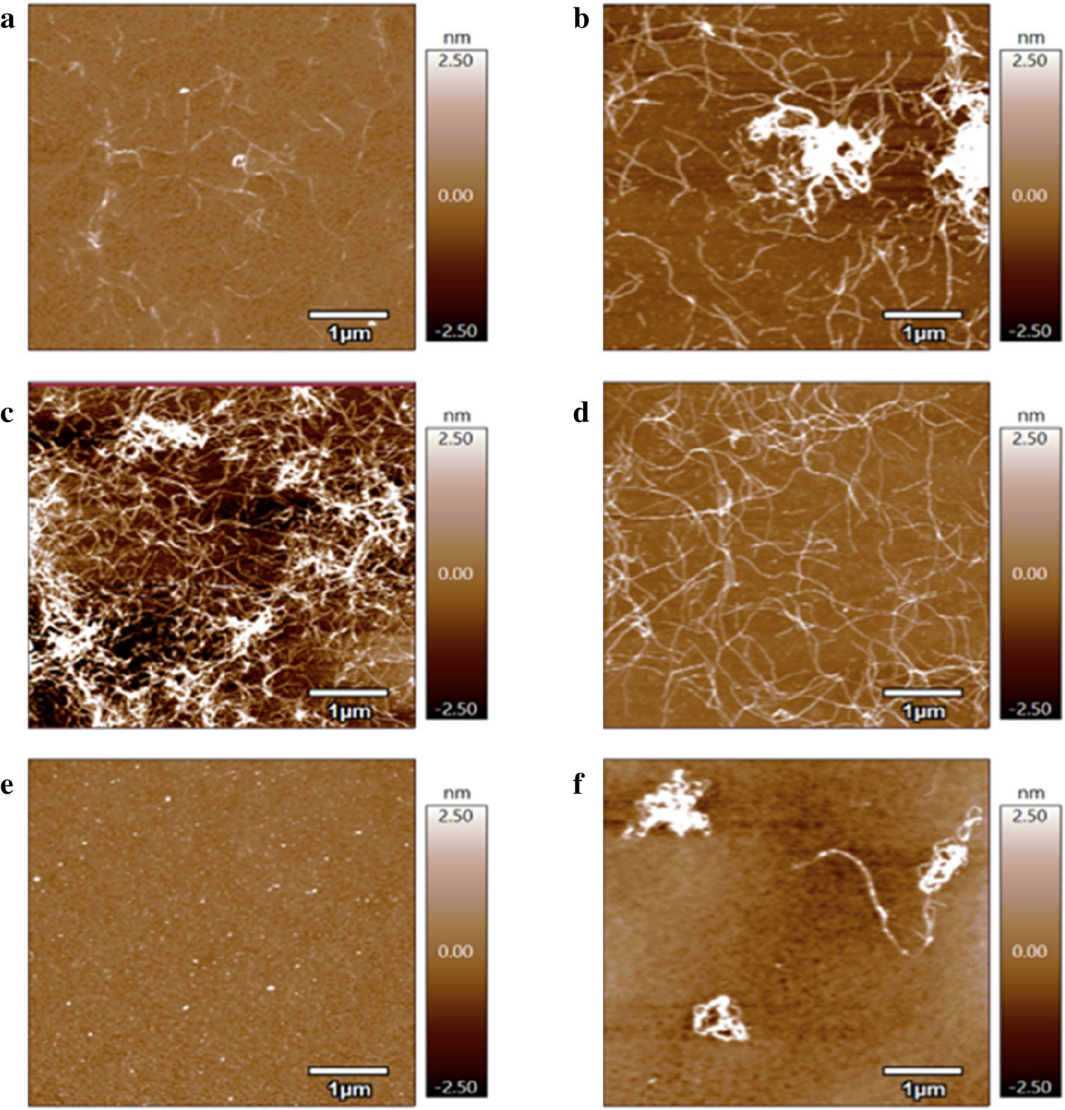
AFM analysis of Aβ aggregation in the presence of HyCar. AFM topography images of Aβ_42_ (15 μM) incubated at 37 °C alone for 0 h **(a)** or 24 h **(b)** or co-incubated for 24 h with 10 µM Hy(200) **(c)**, 0.4 μM Hy(700) **(d)**, 10 μM HyCar(200)14 **(e)** or 0.4 μM HyCar(700)35 **(f)**.

Figure 7 shows the AFM height micrographs of Aβ assemblies for freshly dissolved and 24 h-incubated solutions in MOPS buffer at 37 °C, either in the absence or presence of the different Hy derivatives. A mixed population of small oligomers was found for freshly dissolved Aβ_42_ samples (Fig. 7a). The formation of oligomer species could reasonably be attributed to the interaction of the monomer forms with the mica surface, as previously reported^62^. After 24 h of incubation, bundled assemblies of fibrils were visible for Aβ_42_ alone (Fig. 7b) and Aβ incubated in the presence of Hy(200) (Fig. 7c) or Hy(700) (Fig. 7d). In contrast, an evident fibril antiaggregant effect was detected for Aβ_42_ co-incubated with HyCar conjugates, since Aβ_42_ + HyCar(200)14 samples exhibited only oligomers (Fig. 7e), while few coiled fibrils and oligomers were found for Aβ_42_ + HyCar(700)35 (Fig. 7f).

AFM analyses were also performed to test the capability of the HyCar compounds to dissolve the preformed Aβ fibrils (Supplementary Figure S6). Indeed, for Aβ_42_ alone, after 24 h of ageing after the aggregation period (40 h), amyloid structures were clearly visible, with individual fibrils that formed a tightly packed, highly ordered matrix of twisted fibrils laterally self-associated in large, micrometre-sized aggregates (Supplementary Figure S6a). On the other hand, the preformed Aβ fibrils incubated with HyCar(200)14 (Supplementary Figure S6b) or with HyCar(700)35 (Supplementary Figure S6c) displayed disjointed fibrils and mixed networks/separated fibrils, thus confirming the results obtained by ThT measurements.

### Effect of hyaluronic acids and their Car conjugates on Aβ_42_ cytotoxicity

**Figure 8:**
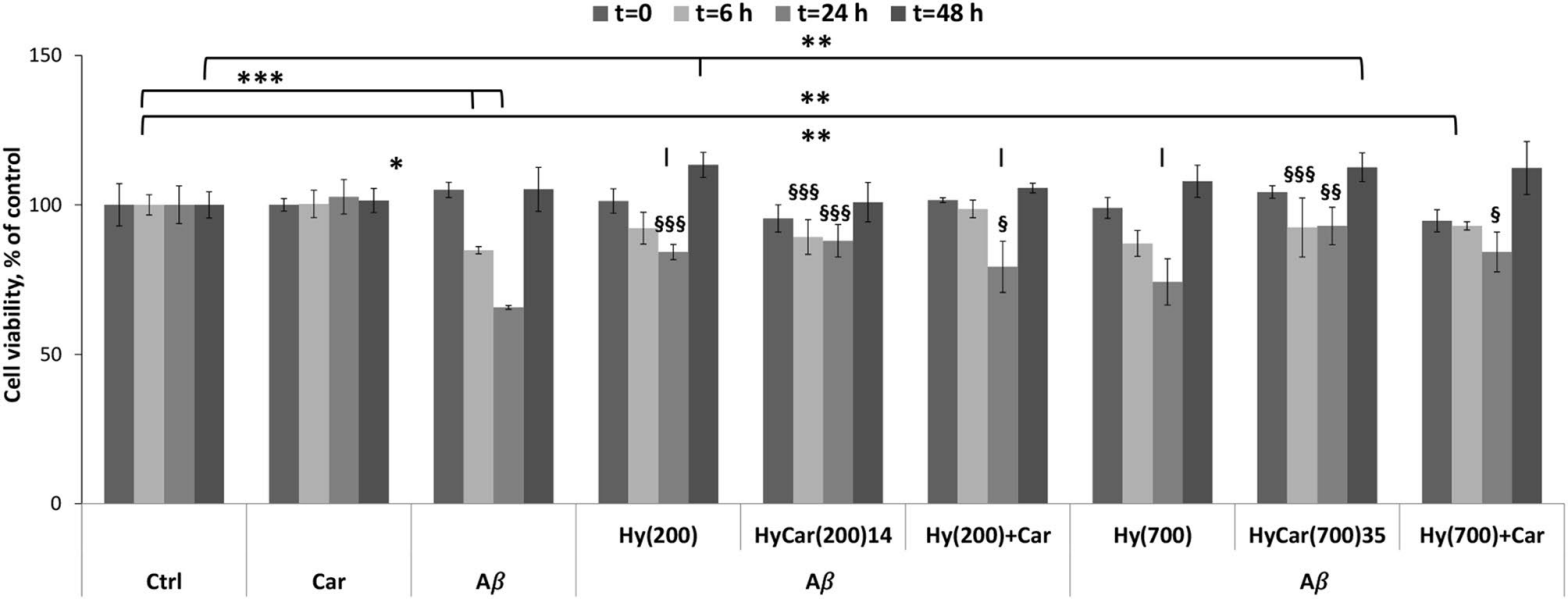
Aβ-induced toxicity in vitro in the presence of HyCar derivatives and their parent compounds. Viability of neuroblastoma SH-SY5Y cells after 24 h of incubation with differently pre-incubated Aβ_42_ (6, 24, 48 h) in the absence or presence of HyCar derivatives (HyCar(200)14, HyCar(700)35), the parent polymeric compounds (Hy(200) and Hy(700)) or a mixture of the latter with Car ((Hy(200) + Car and Hy(700) + Car). The results are presented as the mean ± SD with 3 replicas for each condition (* = p ≤ 0.05; ** = p ≤ 0.005; *** = p ≤ 0.001 with respect to the untreated control; § = p ≤ 0.05; §§ = p ≤ 0.005; §§§ = p ≤ 0.001 with respect to Aβ treatment).

It has been previously shown that oligomeric aggregates of Aβ exhibit profound neurotoxic effects and the ability to form insoluble amyloid deposits in the brain, particularly in the cerebrum and hippocampus^63^. The neurotoxic effects of Aβ_42_ oligomers with both in vitro and in vivo models result in the cognitive deficiencies prevalent in the pathogenesis of AD^64^. Thus, to assess the cytotoxicity of the oligomeric species, the effect of Aβ_42_ at different levels of oligomerization on the viability of undifferentiated SH-SY5Y cells was investigated via the MTT assay. Cells were treated for 24 h with the different compounds in the presence of Aβ_42_ that was pre-incubated for 6, 24 or 48 h (see “Material and methods”). Consistent with previously reported results^65^, we found a decrease in SH-SY5Y cell viability after treatment with 5 μM pre-incubated Aβ for 6 h and 24 h. According to the data obtained from the above ThT assays, showing amyloid aggregate formation after 6 and 24 h of pre-treatment (Supplementary Figure S5), the MTT assay revealed that the presence of exogenous Aβ_42_ oligomers decreased cell viability by 15 ± 1% and 34.3 ± 0.7%, respectively. Notably, freshly dissolved or pre-incubated Aβ_42_ (for 48 h) did not have a significant effect on neuroblastoma cell viability. This suggests the existence of a different Aβ_42_ status during the 48 h of pre-incubation. How HyCar(200)14 and HyCar(700)35 affect Aβ_42_ oligomer-induced neuronal toxicity was examined. The results (Fig. 8) show that Hy(200) and Hy(700) as well as their Car conjugates significantly increase cell viability. This effect could be ascribed to a decrease in the toxic oligomer species that directly reduces the Aβ-induced cell toxicity.

## Discussion

The potential role of Car in brain-related disorders has recently been reviewed^66^, suggesting that reduced plasma Car levels^67^ characterize patients with probable AD, while the carnosinase level has been proposed as a CSF biomarker for early stages of AD^68^. To avoid or delay the carnosinase degradation effect on Car, different derivatives of the dipeptide have been synthesized by conjugating it with derivatives of vitamin E or small carbohydrates^69–71^.

Our findings also show that two Hy chains with different molecular weights (200 and 700 kDa) are able to protect Car from the degradative action of carnosinase when the dipeptide is covalently drafted on the polymer to form HyCar conjugates. In contrast, the mixture of the parent compounds does not prevent or delay the enzymatic degradation of Car.

Polymeric carbohydrates are among the most abundant components of the extracellular matrix, such as the GAG chains in proteoglycans (PGs)^72^. They are associated with different amyloid deposits^73–76^ and are involved in the formation of fibrils. Sulphated GAGs stabilize mature fibrils against dissociation^77^ and prevent their proteolytic degradation^78^; at the same time, sulphated GAGs attenuate the neurotoxic effect of Aβ in cellular assays^79^. AD therapeutic approaches are usually designed to prevent Aβ fibrillogenesis through reduction of Aβ production, inhibition of fibril formation, or removal of fibril aggregates^80^.

It has been suggested that GAG-mediated neuroprotection occurs due to the sequestration of Aβ and increased fibril formation^81,82^, consistent with the hypothesis that the generation of senile plaques in AD characterized by fibrils is a protective response aimed at reducing soluble Aβ neurotoxicity^79^. The promotion of Aβ fibril formation has been attributed to the negative charges of sulphated GAGs^76^ and to other GAGs, including Hy, bearing carboxyl groups instead of sulphate groups^82^. Conversely, some findings indicate that no fibril formation occurs in the presence of Hy^21^.

PGs and Aβs colocalize in senile plaques in the AD brain. MD simulation has shown that the GAGs interact by means of their negative charges with the Aβ domain, characterized by the presence of a basic residue cluster (HHQK) and a contiguous tyrosine, while chitosan, with its positive charges, interacts with the Aβ regions rich in Asp and Glu residues^82^.

It has been suggested that low-molecular-weight heparins can perturb the interactions between PGs and Aβs, blocking or preventing the process of amyloidogenesis^21^. In on-pathway fibrillation, Aβ monomers give rise to fibrils through many metastable steps of oligomeric species formation characterized by β-rich structures^83,84^.

Among natural inhibitors of Aβ amyloid formation, polyphenols such as epigallocatechin-3-gallate (EGCG) and curcumin have been extensively studied^22,85,86^. EGCG could interact with different residues of Aβ_42_ (Phe4, Arg5, Phe19, Phe20, Glu22, Lys28, Gly29, Leu34-Gly37, and Ile41) and form hydrogen bonds with Aβ^85^, while curcumin was able to bind to the 12^th^ and 17^th^ to 21^st^ residues of Aβ_42_^86^.

Mono-curcumine, mono-EGCG and the dual-EGCG-curcumine conjugated with Hy (MW = 1 × 10^6^ kDa) have been synthesized^87,88^. The mono-conjugated derivatives^87^ inhibited Aβ fibril formation more strongly than free polyphenols, while Hy modified with the two polyphenols was more efficient than the single conjugates with EGCG and curcumine^88^. All the modified polyphenols decreased the amyloid cytotoxicity more efficiently when conjugated with Hy. The formation of Hy conjugate nanogels induced an isolation effect, hindering the interactions between Aβ molecules, while hydrophobic interactions of Aβ with EGCG or curcumin and electrostatic repulsion between the bound Aβ and like-charged Hy altered the Aβ monomeric species structures, giving rise to off-pathway aggregation. These two effects were correlated with the conjugated nanostructures that changed with the loading percentage of Hy.

In this context, our results provide evidence that Hy(200) inhibits Aβ_42_ aggregation in a dose-dependent manner (Fig. 5) and protects cells from the toxicity induced by amyloid oligomeric species. Hy(700) induces cell viability in the presence of amyloid species, while it is a modest antiaggregation agent, probably due to the lower concentrations employed (nanomolar range) in the ThT assay than those used for Hy(200) (micromolar range). Furthermore, both the low- and high-molecular-weight hyaluronan chains are able to dissolve fibril formation (Supplementary Figure S3). These protective effects of Hy(200) and Hy(700) found in our experiments are in contrast with those reported in recent years, where the hyaluronic acids did not affect Aβ aggregation and toxicity. The high MW of Hy (1 × 10^6^ Da) employed to examine the Aβ antiaggregation effect of polyphenol conjugates with hyaluronic acids and the related difficulties in handling the nanogels could explain the different findings^87,88^. Moreover, the use of two different sizes of Hy should have an important impact on their potential applications because the biological function of Hy and its derivatives also depends on the MW^89^.

All the HyCar derivatives that we synthesized have a loading percentage of Car higher than the highest substitution degree obtained for the synthesis of other antiaggregant Hy conjugates^88^.

HyCar clearly inhibits the Aβ_42_ aggregation process; this effect increases proportionally with Car loading, influencing both the lag phase and the final growth. Moreover, the HyCar conjugates are more efficient than the parent compounds (individually or as a mixture) in inhibiting Aβ_42_ aggregation, suggesting the existence of a synergistic effect between the polymer and the dipeptide. A prominent role is played by Car, whose antiaggregant properties observed *in vitro*^37,41^ have been attributed to the interaction with the CHD domain^14,15^ and turn region^16,17^. Furthermore, molecular docking^41^, MD simulation, and NMR results^37^ indicated perturbation of the non-covalent interactions between the Aβ side chains of amino acids involved in the intermolecular salt bridge between two Aβ monomers^90^. Moreover, the electrostatic effect has already been proposed to justify the antiaggregant activity of chitosan-hyaluronan nanoparticles towards self-induced amyloid aggregation^91^. Additionally, in the case of the HyCar derivatives, the increase in the number of positively charged sites (imidazole groups) and the decrease in the number of negatively charged carboxylic groups with respect to the unconjugated polymer could account for the antiaggregant activity of the HyCar derivatives being higher than that reported for Hy. The capacity of the Hy scaffold to segregate the Aβ molecules should also be an important aspect that makes the polymer, conjugated or not, more prone than Car to interfere with the on-pathway aggregation of Aβ^88^. Increasing the loading percentage of Car on the Hy scaffold could represent a promising strategy to further improve the antiaggregant properties of the HyCar derivatives, as well the protection against the cytotoxic effects of the amyloid oligomer species.

The His13-Lys16 region, encompassing two histidine residues of the Aβ domain, has been suggested to be involved in the molecular interaction responsible for the inhibitory effect of negatively charged GAG^82^. The same Aβ region is involved in the physiological degradation of Aβ catalysed by IDE^61^. Both IDE and Aβ show conformational transitions during the proteolytic process. An Aβ region switches from an α-helix to a β-strand structure to fit the IDE catalytic site. The same conformational shift accounts for the formation of oligomers and aggregates, both in vitro and in vivo, starting from amyloidogenic substrates. This clearly highlights the potential role of IDE in preventing the aggregation process that originates from the abnormal misfolding of amyloidogenic proteins.

The IDE-mediated hydrolysis of Aβ results in a hydrolytic pattern characterized by a sequential mechanism. In the early stage of hydrolysis, a conserved exosite domain of IDE binds the N-terminal region of Aβ. Therefore, the primary C-truncated Aβ fragments contain no less than 9–10 amino acid residues, and the main targeted regions are those encompassing the hydrophobic Phe residues (Phe19–Phe20), as well as the vicinal His residues (His13–His14). Longer incubation times promote proteolysis on secondary cleavage sites of Aβ, mainly moving close to the primary cleavage sites^46^. For this reason, monitoring the formation of the hydrolytic peptides Aβ_1-13_ and Aβ_1-19_ represents a new and important approach for testing the effects of Hy, Car and HyCar derivatives in the early stage of IDE-mediated Aβ hydrolysis.

The outcome of the IDE-dependent proteolysis assay is further proof of the interaction between the HyCar derivatives and the amyloid peptide. The hydrolytic pattern of amyloid degradation is not markedly modified by HyCar or by the mixture of the parent compounds, thus supporting the non-covalent type of HyCar-Aβ interaction. Moreover, when Car is covalently linked to Hy, the resulting effect on the kinetic trend of the Aβ hydrolytic pattern is significantly different from that exhibited by the Car + Hy mixture. This clearly shows that the chemical conjugation of Car to Hy has a gain-of-function effect on the interaction with Aβ. The synergistic effect between the polymer and the dipeptide, which is associated with the antiaggregant properties of HyCar, also has a clear impact on the Aβ hydrolysis catalysed by IDE. The observed dose-dependent effects of the HyCar conjugate could also be due, at least in part, to the direct effect of viscosity changes that could in turn influence the enzymatic hydrolysis and on-pathway aggregation process of the amyloid peptide.

From AFM analyses, both height and amplitude micrographs of Aβ grown in the presence of the Hy conjugate at low molecular weight demonstrate the capability of the hyaluronic polymer chains to interfere with amyloid fibril formation, thus hindering their aggregation and/or disentangling some previously associated fibrils.

## Concluding remarks

Finding pharmacological approaches that cure or, better, prevent the onset of AD, the most common neurodegenerative disorder, is an important challenge for researchers. Many efforts have recently been devoted to developing multi-target ligands that could counteract the multiple facets of this devastating disorder, such as toxic aggregation, oxidative stress and metal dyshomeostasis. Moreover, investigating the effect of endogenous compounds or a combination thereof is a promising approach to prevent the side effects of entirely synthetic drugs.

The new HyCar derivatives combine the beneficial properties of both hyaluronic acid and Car. The synergistic effect of the parent compounds confers antiaggregant activity towards the amyloid-type aggregation of Aβ. IDE-dependent Aβ degradation, as well as the cytotoxic effect of the oligomeric species, is also affected by the non-covalent interaction with HyCar. This encouraging outcome is also favoured by the resistance of the Car moiety to the action of carnosinase.

Overall, new compounds were obtained by combining the well-known protective features of Car with the ability of Hy to recognize its CD44 receptor, present at high levels in AD brains^92,93^. Therefore, we can speculate that this new class of Aβ aggregation antagonists can pave the way to a possible translational scenario of new HyCar compounds.

## Materials and methods

### Chemicals, reagents and common instrumentation

All solvents and reagents, unless otherwise specified, were purchased from Sigma-Aldrich. Sodium hyaluronate (200 and 700 kDa) was a kind gift from Fidia Farmaceutici S.p. A (IT). 3-Hydroxy,1,2,3-benzotriazin-4(3H)-one (HOOBt) and trifluoroacetic acid (TFA) were purchased from AAPPTec and VWR, respectively. 3-(*N*-morpholino)-propanesulphonic acid (MOPS) buffer solution (1 mM, pH 7.4, supplemented with 0.003 M KCl and 0.14 M NaCl) was prepared from a powder (Amresco Biochemicals).

Anhydrous methanol was kept for 24 h on 4 Å molecular sieves before use. Carnosine methyl-ester (Car-OMe) was synthesized in anhydrous methanol, as previously reported^94^.

All the fluorescence and UV–Vis spectra were reordered by using a multimode microplate reader (Varioskan Flash, Thermo Scientific).

### Synthesis of the HyCar derivatives

To synthesize HyCar(200)35, sodium hyaluronate (200 kDa, 1 g) was added, under stirring, to 20 ml of cold tetrahydrofuran (THF). The resulting suspension was added to 2.6 mmol of HOOBT in 20 ml of H_2_O/THF (1:1 v/v), 1.56 mmol of Tris[2-(2-methoxyethoxy)ethyl]amine in 10 ml of H_2_O/THF (1:1 v/v), and 1.3 mmol of Car-OMe in 5 ml of methanol. After 30 min at 5 °C, *N*-(3-dimethylaminopropyl)-*N*'-ethyl-carbodiimide hydrochloride (EDC*HCl; 1.3 mmol) in 10 ml of water was added to the mixture. After 20 h at 5 °C under stirring, the reaction mixture was treated with 0.1 N NaOH (100 ml) for 3.5 h at 5 °C. The pH was then adjusted to 7.0 with 2 N HCl, and the conjugate was precipitated by the addition of acetone (800 ml). After centrifugation, the product was dissolved in water and dialyzed for 60 h. The conjugate was then recovered and lyophilized (yield 75%).

^1^H-NMR (D_2_O, 500 MHz) (ppm): 8.66, s, (H-2 of the imidazole ring); 7.34, s, (H-5 of the imidazole ring); 4.61–4.52, m (broad), (H-6 of glucuronic acid residues, H-1 of N-acetylglucosamine residues and methyne of histidine); 3.90–3.23, m (broad), (H-2, H-3, H-4 and H-5 of the glucuronic acid residues; H-2, H-3, H-4, H-5, and H-6 of the *N*-acetyl glucosamine residues; and β-methylene of the β-alanyl residues); 3.17, m, (methylene of histidine); 2.44, m, (α-methylene of the β-alanyl residues); 2.06, s (broad), (methyl of the *N*-acetylglucosamine residues).

The amount of Car linked to hyaluronic acid was determined from the ratio between the integration value of the signal at 2.06 ppm (related to the acetyl groups of Hy) and that of the H-2 or H-5 signal of the imidazole ring of histidine (at 8.66 and 7.34 ppm, respectively).

For the synthesis of HyCar(200)7,10,14, the procedure was not different from that adopted to synthesize HyCar(200)35, except for the amounts of Car-OMe and EDC:

- for HyCar(200)7: Car-OMe (0.26 mmol) and EDC*HCl (0.26 mmol);
- for HyCar(200)10: Car-OMe (0.39 mmol) and EDC*HCl (0.39 mmol);
- for HyCar(200)14: Car-OMe (0.52 mmol) and EDC*HCl (0.52 mmol).

Finally, HyCar(700)35 was synthesized as described for the production of HyCar(200)35, except for a few aspects: the volume of the solvents was doubled, and 20 ml of water was added to the final reaction mixture.

### Molecular weight distribution and intrinsic viscosity of the HyCar conjugates

The medium molecular weight of each conjugate was determined by means of size exclusion chromatography on a Visctek GPCmax VE 2001 chromatographic system (Malvern) equipped with two TSK-GEL GMPWXL columns (7.8 mm ID × 300 mm; TOSOH BIOSCIENCE) installed in series. The system was coupled with three detectors placed in series: a refraction index detector, a light scattering detector and a four-capillary differential viscometer. Each sample was eluted using 0.1 M sodium nitrate in water containing 0.5 g/L sodium azide with a flow rate of 0.6 ml/min at 40 °C. Omnisec 4.1 software (Marvern Panalytical, https://www.malvernpanalytical.com/en/products/product-range/viscotek-range/viscotek-systems/viscotek-tdamax/accessories/omnisec-software) was used for data acquisition and analysis. Triple-detector detection was performed on a Viscotek TDA 302 (Malvern).

### Enzymatic hydrolysis by carnosinase

The time-dependent stability of the conjugate HyCar(200)7 towards CN1, compared to that of the parent compounds, was determined by incubating these substances (900 μM) in Tris/HCl buffer (50 mM, pH 8.0) at 37 °C with the enzyme, purified from the culture medium of stably transfected HeLa cells, as previously reported^57^. Several aliquots were sampled until 5 h, and the reaction was stopped by adding trichloroacetic acid (TCA). The amount of histidine was determined after a reaction with ortho-phthalic aldehyde (OPA; Fluka) by means of a fluorometric assay^60^, adapted to multiwell plate detection^70^.

### Aβ_42_ sample preparation

Monomeric Aβ_42_ was obtained using a variant of Zagorsky’s protocol^95–97^. Briefly, 1 mg of peptide was dissolved in TFA (1 ml). The solution was sonicated for 10 min, and TFA was then evaporated under a gentle stream of N_2_. One millilitre of HFIP was added to the peptide. After 1 h of incubation at 37 °C, the peptide solution was dried under a stream of N_2._ This procedure was repeated 2 times. The dried peptide was then dissolved in HFIP (1 ml) and fast-frozen at -80 °C before lyophilization (10 h). The lyophilized sample was re-suspended in 5 mM NaOH solution in ultrapure water (Millipore, 18.2 MΩ·cm resistivity at 25 °C) to a final concentration of 1.5 mM and stored at − 80 °C.

### Aβ aggregation assay

The effect of the compounds of interest on the formation of amyloid-type fibrils of Aβ has been assayed as previously reported^98^. Briefly, all the HyCar derivatives (or their parent compounds), Aβ_42_ (15 µM)and ThT (45 µM) were incubated in MOPS buffer (50 mM, pH 7.4) at 37 °C in the multiplate reader. The collected fluorimetric data (excitation and emission wavelengths were 450 nm and 480 nm, respectively) of all the measurements, carried out in triplicate, were fitted to Eq. (1).

1 $$F\left(t\right)={F}_{0}+\frac{{F}_{max}-{F}_{0}}{1+{e}^{-\frac{t-{t}_{1/2}}{k}}}$$

*F*_*max*_* – F*_*0*_ is the maximum fluorescence gain due to the amyloid aggregation value, whereas the lag phase (*t*_*lag*_), calculated by using Eq. (2), represents the time interval before the formation of ThT-sensitive amyloid species.

2 $${t}_{lag}={t}_{1/2}-2/k$$

The parameters of each set of measurements were expressed as the mean ± SD.

### Aβ disaggregating assay

Solutions containing Aβ_42_ (15 µM) and ThT (45 µM) were incubated in MOPS buffer (50 mM, pH 7.4) with a black 96-well plate (Nalge-Nunc, Rochester, NY). After 40 h at 37 °C, the compounds of interest were added into the solutions, and the plates were kept at 37 °C for 24 h in a multiplate plate reader. Amyloid disaggregation was followed by measuring the ThT fluorescence emission at 480 nm after excitation at 450 nm. Aβ dissolution was calculated as the percent decrease in ThT after incubation with the compounds of interest.

### Enzymatic hydrolysis of Aβ

The amyloid peptide Aβ_1–28_ (10 µM), HyCar(700)35 (0.1 or 0.4 µM), its parent compound (Hy and Car, 0.4 µM and 270 µM, respectively) or a mixture of the parent compounds (Hy + Car, 0.4 µM and 270 µM, respectively) was incubated at 37 °C in MOPS buffer (1 mM, pH 7.4) in the presence of IDE (Sigma-Aldrich) (10 nM) for 40 min. The reaction was stopped with 0.5% TFA and diluted 1:10 with water. The hydrolytic peptides were analysed by a MALDI TOF/TOF 5800 Analyzer (AB SCIEX, Foster City, CA) by using previously reported instrument settings^99^. A saturated solution of α-cyano-4-hydroxycinnamic acid (CHCA) in water:acetonitrile 2.3:1 with 0.3% TFA was used as the matrix. Each sample was spotted three times, and four spectra were acquired for each spot, thus obtaining a set of twelve spectra for each sample.

### AFM measurements

The stored Aβ_42_ solution was diluted to 15 μM in MOPS buffer (1 mM, pH 7.4) containing Hy200, Hy200 supplemented with Car (1.84 mM), HyCar35 (200), Hy700, Hy700 supplemented with Car (0.26 mM), HyCar35 (700) and Car (1.84 mM) at a hyaluronic acid concentration of 10 μM for the low molecular weight (200 kDa) and 0.4 μM for the high molecular weight (700 kDa). All the samples were incubated in an orbital shaker at 37 °C and 190 r.p.m. for 24 h. To assess the effect of hyaluronic acid on Aβ_42_ aggregation, each measurement was carried out immediately after dilution of the peptide and after 4 and 24 h of incubation.

To register AFM images, we used a previously described procedure^96^. Briefly, 10 μL of each sample was placed on freshly cleaved muscovite mica (Ted Pella, Inc.) and incubated for 5 min at room temperature. Then, samples were washed with 1 ml of ultrapure water, dried under a gentle nitrogen stream and immediately imaged. A Cypher AFM instrument (Asylum Research, Oxford Instruments, Santa Barbara, CA) operating in tapping AC-mode was used. Tetrahedral tips made of silicon and mounted on rectangular 30-μm-long cantilevers were purchased from Olympus (AT240TS, Oxford Instruments). The probes had nominal spring constants of 2 N/m and driving frequencies of 70 kHz. Images were scanned, and the sizes of the aggregates were measured using a free tool in the Asylum Research offline section analysis software program.

### Cell culture maintenance and treatments

Human neuroblastoma (SH-SY5Y line) cells were treated as previously reported^100^. Briefly, cells were cultivated for no more than 20 passages in full medium, i.e., Dulbecco’s modified Eagle’s medium (DMEM)/Ham’s F-12 medium (F12) supplemented with 10% foetal bovine serum (FBS), 2 mM l-glutamine and 100 μg ml^−1^ streptomycin. The cell culture was grown in tissue culture-treated Corning flasks (Sigma-Aldrich, St. Louis, MO) in a humidified atmosphere (5% CO_2_) at 37 °C (Heraeus Hera Cell 150C incubator).

### Cytotoxicity assays

The MTT method with the undifferentiated SH-SY5Y cell line was employed for the cell viability assays. The day before the experiment, cells were seeded at a density of 2.5 × 10^4^ cells per well (50% confluence) in full medium on Corning tissue culture treated 48-multiwell plates (Sigma-Aldrich, St. Louis, MO) until cellular adhesion was attained. Samples of Aβ_42_ (15 µM) were pre-incubated for 6, 24 and 48 h at 37 °C alone or in the presence of HyCar(200)14, HyCar(700)35, Hy(200) and Hy(700) with or without Car. Cells were treated for 24 h with pre-incubated Aβ_42_ (5 µM) in the presence of the studied compounds in full medium supplemented with 1% FBS. Then, cell viability was determined at 37 °C by the 3-(4,5-dimethylthiazol-2-yl)-2,5-diphenyltetrazolium bromide (Sigma-Aldrich, St. Louis, MO) method (MTT assay) as previously described^101^. After 90 min, the formazan salts produced by succinate dehydrogenase activity in live cells were solubilized by the addition of DMSO, and absorbance was measured at 570 nm by a microplate plate reader. The results were expressed as % of viable cells. At least three replicate experiments were conducted, and the averaged data with standard deviations were reported. Student's t-test was performed for statistical comparisons to analyse the variance, and a p-value less than 0.05 was considered to be statistically significant.

## Supplementary information

Supplementary Information.
