# Supplementary material for: Hyaluronan-carnosine conjugates inhibit Aβ aggregation and toxicity
Source: Sci Rep. 2020 Sep 29;10:15998. doi: 10.1038/s41598-020-72989-2 (PMC7524733; doi:10.1038/s41598-020-72989-2)
Supplement: Supplementary file 1 — Supplementary Information. [file 41598_2020_72989_MOESM1_ESM.docx]

**Hyaluronan-carnosine conjugates inhibit Aβ aggregation and toxicity**

Valentina Greco,^1^ Irina Naletova,^2^ Ikhlas [M M Ahmed](https://pubmed.ncbi.nlm.nih.gov/?term=Ahmed+IMM&cauthor_id=30996991),^3^ Susanna Vaccaro,^4^ Luciano Messina,^4^

Diego La Mendola,^5^ Francesco Bellia,^3*^ Sebastiano Sciuto,^1*^ Cristina Satriano,^1^ Enrico Rizzarelli^1,2,3^

*^1^ Department of Chemical Sciences, University of Catania, A. Doria 6, 95125 Catania (Italy)*

*^2^Consorzio Interuniversitario per lo Studio dei Metalli nei Sistemi Biologici, C. Ulpiani, 27 –70126 Bari (Italy)*

*^3^Institute of Crystallography, CNR, P. Gaifami 18, 95126 Catania (Italy)*

*^4^Fidia Farmaceutici, 96017 Noto (SR)* (*Italy*)

*^5^Department of Pharmaceutical Sciences, University of Pisa, Bonanno Pisano 12, 56126 Pisa (Italy)*

**Supplementary information**

**Table S1**. Mechanical and structural characterization of the HyCar derivatives. Medium Molecular Weight (MMW) and intrinsic viscosity (I.V.) values, obtained for each synthesized conjugates (HyCar(200)35, HyCar(200)14, HyCar(200)10, HyCar(200)7 and HyCar(700)35) as well as for the free polysaccharide (Hy(200) and Hy(700)). Data acquisition and data analysis were performed by using Omnisec software (version 4.1, Marvern Panalytical, https://www.malvernpanalytical.com/en/products/product-range/viscotek-range/viscotek-systems/viscotek-tdamax/accessories/omnisec-software)

|  | **MMW (kDa)** | **I.V. (g/dL)** |
| --- | --- | --- |
| *Hy(200)* | 193 | 5.2 |
| *HyCar(200)7* | 200 | 4.8 |
| *HyCar(200)10* | 229 | 5.0 |
| *HyCar(200)14* | 236 | 4.9 |
| *HyCar(200)35* | 248 | 4.5 |
| *Hy(700)* | 745 | 17.4 |
| *HyCar(700)35* | 855 | 9.4 |

**Table S2**. Kinetic measurements of the amyloid aggregation in the presence HyCar(200) derivatives. Kinetic parameters related to the aggregation of Aβ_1–42_ alone (CTRL), incubated with the Hy(200) derivatives of Car (Hy(200)Car7, Hy(200)Car10, Hy(200)Car14) or their parent compounds (Hy(200), Car or a mixture of them), the concentrations of the tested compounds being 10 µM (Hy(200), Hy(200)Car7, Hy(200)Car10, Hy(200)Car14) or concern 700 µM (Car). The concentration values of Hy(200) and Car, both separately and in a mixture, are equivalent to the content of Hy and Car units in the sample containing HyCar(200)14.

|  | **CTRL** | **Hy(200)** | **Car** | **Hy(200)+Car** | **HyCar(200)7** | **HyCar(200)10** | **HyCar(200)14** |
| --- | --- | --- | --- | --- | --- | --- | --- |
| ***F_max_ - F_0_*** | 51.2 ± 0.9 | 17.4 ± 0.7 | 50.8 ± 0.7 | 16.4 ± 0.9 | 17.9 ± 0.8 | 19.4 ± 0.5 | – |
| ***t_lag_*** | 5.9 ± 0.2 | 5.7 ± 0.6 | 5.6 ± 0.3 | 5.5 ± 0.4 | 6.3 ± 0.4 | 8.9 ± 0.4 | – |

**Table S3**. Kinetic measurements of the amyloid aggregation in the presence HyCar(200)35. Kinetic parameters related to the aggregation of Aβ_1–42_ alone (CTRL), incubated with Hy(700), Car, a mixture of them (Hy(700)+Car) or the Hy-Car derivative (HyCar(700)35), the concentrations of the tested compounds being 0.40 µM (Hy(700), HyCar(700)35) or 270 µM (Car). The concentration values of Hy(700) and Car, both separately and in a mixture, are equivalent to the content of Hy and Car units in the sample containing HyCar(700)35.

|  | **CTRL** | **Hy(700)** | **Car** | **Hy(700)+Car** | **HyCar(700)35** |
| --- | --- | --- | --- | --- | --- |
| **F_max_ - F_0_** | 8.8 ± 0.5 | 7.9 ± 0.6 | 8.9 ± 0.6 | 7.5 ± 0.2 | 4.4 ± 0.2 |
| ***t_lag_*** | 9.8 ± 0.3 | 10 ± 0.2 | 9.5 ± 0.4 | 9.5 ± 0.4 | 24.5 ± 0.8 |

**Figure S1.** Carnosinase-mediated hydrolysis of Car and HyCar(200)35. The fluorescence intensity (proportional to the histidine content) is reported over reaction time.

**Figure S2**. Kinetic trends of the amyloid aggregation in the presence HyCar(700)35. Kinetic profiles of Aβ_1–42_ aggregation alone (CTRL, 15 µM) or incubated with the HyCar(700)35 (20-400 nM).

**Figure S3**. Effect of HyCar derivatives and their parent compounds on the dissolution of pre-formed Aβ fibrils. ThT decrease after the incubation of preformed Aβ fibrils (15 µM) with HyCar(200)14 (10 µM), HyCar(700)35 (0.4 µM) or their parents compounds: Hy(200) (10 µM), Hy(700) (0.4 µM), Car (420 or 360 µM), and mixtures of them.

**Figure S4**. Kinetic trend of the Aβ hydrolytic pattern in the presence of HyCar(700)35 or its parent compounds. Time-dependent hydrolytic pattern of the IDE-mediated degradation of Aβ_1–28_ alone (CTRL) or in the presence of: HyCar(700)35 (0.1 and 0.4 µM) Hy(700) (0.4 µM), Car (270µM) or a mixture of Hy and Car (Hy + Car, 0.4 and 270 µM, respectively). The concentration values of Hy and Car, both separately and in a mixture, are equivalent to the content of Hy and Car units in the sample HyCar(700)35 0.4 µM.

| **a**   |
| --- |
| **b**   |
| **c**   |

**Figure S5**. Kinetic trends of the amyloid aggregation in the presence of HyCar derivatives. Kinetic profiles of Aβ_1–42_ aggregation alone (CTRL, 15 µM), incubated with the (a) HyCar(200)14 or their parent compounds (10 µM), (b) HyCar(200)14 (1-10 µM) and (c) Hy(700) derivatives of Car or their parent compounds (10 µM). Arrows indicate the reaction samples (0, 6, 24 and 48 h) used for AFM analysis and measurements on cell lines.


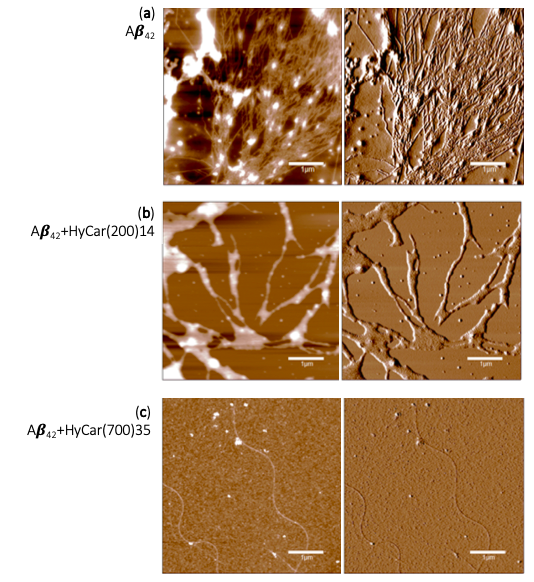


**Figure S6.** AFM analysis of preformed Aβ fibrils in the presence of HyCar. AFM images of topography (left panel) and amplitude (right panel) for 15 μM Aβ_42_ after 72 h incubation in MOPS buffer at 37°C, either alone (a) or in the presence of 10 μM HyCar(200)14 (b) or 0.4 μM HyCar(700)35 (c). z scale = 20 nm.
